# The importance of ubiquitination and sumoylation on the transforming activity of HTLV Tax-1 and Tax-2

**DOI:** 10.1186/1742-4690-9-103

**Published:** 2012-12-07

**Authors:** Linda Zane, Kuan-Teh Jeang

**Affiliations:** 1 Molecular Virology Section, Laboratory of Molecular Microbiology, the National Institutes of Allergy and Infectious Diseases, the National Institutes of Health, Bethesda, MD, 20892-0460, USA

**Keywords:** HTLV-1, HTLV-2, Tax, ATL, Cell transformation, NF-κB, Sumoylation, Ubiquitination

## Abstract

Human T-cell Leukemia Virus type 1 (HTLV-1) and 2 (HTLV-2) are two closely related human retroviruses. HTLV-1 is associated with an aggressive Adult T-cell Leukemia (ATL) while there is no evidence for an association of HTLV-2 with any human malignancies. The two viruses encode transactivator proteins, Tax-1 and Tax-2 respectively. In ATL, Tax-1 is thought to play a central role in the transformation of a normal T-cell into a leukemic cell; however, it has not been entirely clear how post-translational modifications of Tax-1 influence its transforming activity. Here, we discuss three recent papers that report on the ubiquitination and sumoylation of Tax-1 and Tax-2. We comment on their divergent findings implicating the importance (or lack of importance) of these modifications and other events on Tax activation of NF-κB as related to cellular transformation.

## Background

Human T-cell Leukemia Virus type 1 (HTLV-1) and type 2 (HTLV-2) belong to the deltaretrovirus family. Although these two human retroviruses are closely related, they show different tropism and differential pathogenicity. HTLV-1 preferentially engenders the proliferation of CD4^+^ T-cells while HTLV-2 preferentially performs similarly for CD8^+^ T-lymphocytes [1]. Although first identified in hairy T-cell leukemia, HTLV-2 is currently not believed to be associated with any neoplasia. By contrast, the association between HTLV-1 and Adult T-cell Leukemia has been well established for more than 30 years [2]. HTLV-1 is also the etiological agent of inflammatory diseases such as HTLV-1 associated Myelopathy (HAM)/Tropical Spastic Paraparesis (TSP) [3], uveitis, infective dermatitis and myositis. Both viruses encode a *tax* gene in their respective pX open reading frame located in the 3’ region of the viral genome. HTLV-1 Tax (Tax-1) and HTLV-2 Tax (Tax-2) share amino acid similarities of 85% but differ from each other in many aspects including their cell transforming capacity [4].

A key factor implicated in inflammation and cellular proliferation is NF-κB, which is constitutively active in most tumor cells and is a major survival factor engaged by HTLV-1; suppression of NF-κB has been reported to inhibit the growth of leukemic cells [5]. Tax-1 activation of NF-κB has been regarded as a key event in HTLV-1 transformation. Because HTLV-2 has no malignant association, a longstanding question has been whether Tax-2 is able to activate NF-κB similarly or differently from Tax-1. If both Tax proteins are able to activate NF-κB, what then are the nuanced differences between the mechanisms that specify their activity and/or the magnitude of their activating capacity?

### New findings

Three new reports have added insights to our views on Tax-NF-κB activation. First, in a September 2012 *Retrovirology* paper, Bonnet *et al.* showed that Tax-1 sumoylation and nuclear body formation are not needed for Tax-1 activation of NF-κB [6]. They used a Tax-1 mutant, Tax-P79AQ81A, defective for nuclear body formation. Tax-1-P79AQ81A and wild-type Tax are ubiquitinated at similar levels but mutation of the P79 and Q81 residues dramatically reduced the conjugation of Tax-1-P79AQ81A to endogenous SUMO compared to wild-type Tax-1. Low sumoylation status of this mutant did not prevent its transactivation of a NF-κB promoter, suggesting that sumoylation is not required for Tax-1-induced NF-κB activation. In contrast to sumoylation, the authors showed Tax-1 ubiquitination is important for Tax-1-induced NF-κB activation. The ubiquitinated form of Tax-1 binds to IKKγ/NEMO and triggers IKK activation. Moreover, they observed a correlation between the amount of endogenous phospho-IKKα/β co-immunoprecipitated with Tax-1 and the level of Tax-1 ubiquitination but not with the level of Tax-1 sumoylation.

Second, in a November 2012 *Journal of Virology* paper [7], Journo *et al.* demonstrated by comparing ubiquitination, sumoylation and acetylation modifications that the NF-κB activating functions of Tax-1 and Tax-2 are regulated through distinct molecular mechanisms. Indeed, in contrast to Tax-1, Tax-2 conjugation to endogenous SUMO and ubiquitin on its lysine residues or N-terminal residues was barely detectable, while Tax-2 was acetylated. These low levels of conjugation to endogenous ubiquitin and SUMO did not prevent Tax-2 activation of a NF-κB-dependent promoter or its interaction with IKKγ/NEMO; and the acetylation status of Tax-2 did not affect its ability to activate NF-κB. Furthermore, a lysine-less Tax-2 mutant, which is not ubiquitinable, not sumoylable and not acetylable, is still able to transactivate a NF-κB-dependent promoter and bind and activate the IKK complex to induce RelA/p65 nuclear translocation. Altogether, these data suggest that in contrast to Tax-1, sumoylation and ubiquitination are not essential for Tax-2 to activate NF-κB. Finally, the authors also described, through the use of chimeric proteins containing domains from both Tax-1 and Tax-2, that subcellular localization alone does not account for the low levels of Tax-2 ubiquitination and sumoylation. However, the amino acid context of the targeted lysine residues seems to be critical to the process of ubiquitination and sumoylation of Tax proteins.

Third, Turci *et al.* in the accompanying Retrovirology paper suggest a contrasting view [8]. They report that Tax-1 and Tax-2 share a common mechanism of NF-κB activation; both are dependent on their ubiquitination and sumoylation status. Thus, they show that patterns and levels of ubiquitination between Tax-1 and Tax-2 are conserved, except for a reduced representation of the Tax-2 mono-ubiquitinated form compared to Tax-1. By using Tax-2 and Tax-1 lysine to arginine substitution mutants, they demonstrated that lysine usage for sumoylation differs between Tax-1 and Tax-2. Indeed, contrary to Tax-1, the central lysine residues other that K7 and K8 can be sumoylated in Tax-2. Importantly, Turci *et al.* found that neither Tax-1 nor Tax-2 sumoylation and ubiquitination deficient mutants could activate NF-κB. The authors could, however, restore NF-κB transcriptional activity by fusing ubiquitin or SUMO-1 to the C-terminus of these mutants, suggesting that similar to Tax-1, ubiquitination and sumoylation are needed for Tax-2 transactivation of a NF-κB-dependent promoter. Intriguingly, they also found a direct correlation between the ubiquitination of Tax-1 or Tax-2 and the translocation of RelA to the nucleus as well as between the sumoylation of Tax-1 or Tax-2 and the formation of punctate nuclear structures containing RelA and p300 with Tax-1 or Tax-2.

### Controversies

These three recent studies present interesting comparisons and contrasts. Journo *et al.* reject the involvement of ubiquitination and sumoylation on Tax-2 activation of NF-κB; Bonnet *et al.* refute the requirement of the sumoylation in Tax-1 activation of NF-κB; and, Turci *et al.* propose that both ubiquitination and sumoylation are important to the activation of NF-κB by Tax-1 and Tax-2. How does one move forward and reconcile these discrete results?

There is a large discrepancy between Bonnet *et al.* who concluded that Tax-1 ubiquitination, but not Tax-1 sumoylation, is required for Tax-1 activation of NF-κB and Turci *et al.* who found that both ubiquitination and sumoylation of Tax-1 and Tax-2 mediate their NF-κB activity. The strength of Bonnet’s study is that it was performed in suspension CD4^+^ primary T lymphocytes, the real target cells of HTLV-1 infection *in vivo,* while Turci *et al.* and Journo *et al.* performed most of their experiments in HeLa and 293T attached cell lines. Nevertheless, the absence of correlation established by Bonnet *et al.* between Tax-induced NF-κB promoter activation and Tax-1 sumoylaton is not sufficient to conclude that sumoylation is not required for Tax-1 activation of NF-κB. Indeed, the authors cannot rule out that a low, poorly detectable, sumoylation level of their Tax-1-P79AQ81A mutant may be sufficient to activate NF-κB, given that the biological consequences of conjugation do not appear proportional to the small fraction of substrate that is modified [9]. Likewise, they cannot exclude the possibility that small localized clusters of Tax-1 in HTLV-1 infected cells not detected by their confocal microscopy are present and required for the stimulation of NF-κB transcriptional activity. Finally, the deficit of nuclear body formation in Tax-1-P79AQ81A transfected cells may not be attributed directly to a lower sumoylation level of this mutant, but could rather be due to lowered expression of this mutant.

On the other hand, Turci *et al.* performed all their experiments under conditions where ubiquitin or SUMO proteins were over-expressed, which could lead to inaccurate conclusions from over expression-elicited non-physiological effects [10]. The concern regarding over-expression artifacts perhaps motivated Journo *et al.* and Bonnet *et al.* to perform all their experiments employing conditions of endogenous ubiquitin and SUMO. Furthermore, the key argument of Turci *et al.* establishing the requirement of Tax-1 and Tax-2 sumoylation for their NF-κB activity was based on the results presented in their figure 5. However, the levels of wild type Tax and mutant Tax proteins in their lysates, which were ascertained by Western blot analysis using Tax-1 and Tax-2 antibodies, were not comparable. The expression levels of Tax mutants were lower than those of wild type proteins which could explain reduced NF-κB activity. This issue was addressed in the work of Journo *et al.* who transfected two fold less wild type DNA than mutant DNA to ensure equivalent expression levels. Finally, a strength of the Journo *et al.* work was that they used endogenously expressed ubiquitin and SUMO proteins and adjusted their experimental conditions to achieve similar expression levels of wild type and mutant Tax proteins. Nonetheless, the interpretation of their results could benefit from added statistical rigor. For example, in the comparison of NF-κB luciferase activity between Tax-2 wild type and Tax-2 lysine-less mutant protein in figures 2C, E and G, they used an unpaired *t*-test when a paired *t*-test would have been more appropriate. Potentially, this alternative statistical analysis could explain the lack of significance in their results, which led them to conclude on an absence of correlation between Tax-2 ubiquitination and/or sumoylation with its NF-κB activity.

## Conclusions and futures directions

**Figure 1:**
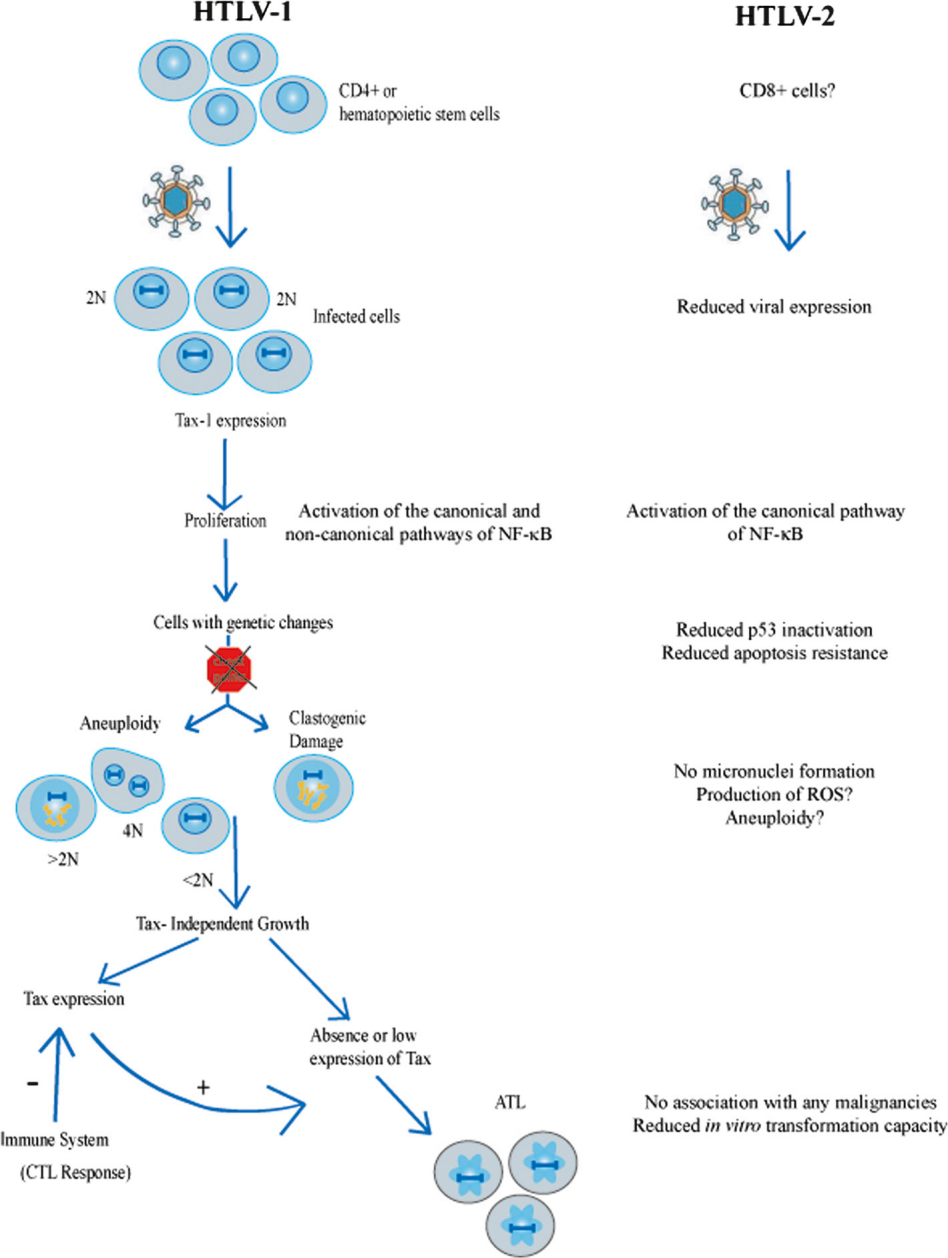
**A schematic representation of putative multi-stepped process of HTLV-1 induced transformation.** Differences between HTLV-1 (left) and −2 (right) in the various steps are outlined.

Prior to the studies of Journo *et al.* and Turci *et al.* few details were understood about the mechanism by which Tax-2 activates the canonical NF-κB pathway. However, like that for Tax-1, the involvement of sumoylation and ubiquitination in Tax-2 activation of NF- κB remains debatable despite these two published works. Congruence in experimental conditions needs to be achieved before one can firmly compare the two studies and adjudicate on the importance of these post-translational modifications for physiological Tax-2 activities. However, for Tax-1, the consensus opinion is that ubiquitination is likely involved in its NF-κB activity [11]. Lastly, one should be duly circumspect about what these three recent papers do not touch upon. Indeed, it is already well-characterized that Tax-1 is able to activate both the canonical and the non-canonical NF-κB pathways while Tax-2 can only activate the former. Even though NF-κB is a major survival factor engaged by HTLV-1, the overall route to ATL malignant transformation is considerably more complex and involves a multi-step process [12] (Figure 1) that also encompasses events such as clastogenic DNA damage [13] and aneuploidy [14], which are not easily explained by NF-κB activation. Thus a more complete picture on transformation would benefit from further comparisons of Tax-1 and Tax-2 for their respective effects on DNA damage, aneuploidy, and *in vivo* proliferation [15]. Nonetheless, although the current three comparative studies on Tax-1 and Tax-2 and NF-κB activation, in many ways raised more questions than answers, they are a step in the right direction. Future comparisons between Tax-1 and Tax-2 on other activities needed for cellular transformation promise to inform and challenge investigators in the coming years.

## Competing interests

The authors have declared that no competing interests exist.

## Authors’ contributions

LZ and KTJ conceived and wrote this Viewpoint together. Both author read and approved the final manuscript.
